# Notes and News

**Published:** 1903-11-07

**Authors:** 


### NOTES AND NEWS.

MR. GEORGE HERRING has fulfilled his promise to add five shillings to every pound collected by the Metropolitan Hospital Sunday Fund this year, and has sent £12,312 to the Fund.

The Lord Mayor and Lady Mayoress unveiled on Monday the tablets over the 15 beds founded in the London Hospital in honour of the visit of the King and Queen during the summer. Each of the beds has been endowed by a separate donor with a sum of £1,000.

THE London County Council's new epileptic colony buildings at Epsom are completed, but they cannot be opened owing to difficulties with the Urban District Council arising out of the question of sewage disposal. The Local Government Board has been appealed to.

THE Lord Mayor and the Lady Mayoress, together with the Sheriffs and their wives, visited the London Temperance Hospital in State last week, to inaugurate a scheme for rebuilding the out-patients' department at a cost of £10,000. It was mentioned that King Edward's Hospital Fund had already contributed £1,500 for the purpose.

ONE lesson, which apparently admits of no contradiction, has been learnt by means of recent inquiry into the main causes of tuberculosis in lunatic asylums. This is that infection plays a very definite part in the spread of disease, and it is to be hoped that this fact will be recognised generally and segregation universally adopted.

THE awakening of the post-graduate is rapidly becoming more visible, and we hope that the stigma of indifference to progress in matters professional will soon pass away from the general practitioner. In connection with the public health laboratories of Owens College a class of local medical men from Burnley and the neighbourhood has been formed to study practical bacteriology. The meetings are weekly.

THE notification of tuberculosis has been made compulsory in Vienna.

A NEW sanatorium for tuberculosis has been opened at Ambrock in Westphalia.

HONG KONG has been declared free of the plague from October 27th. The last weekly record of plague in Mauritius reported 117 cases.

MR. THOMAS BARNARD, of Cople House, Beds, hon. treasurer, has presented a Finsen Light apparatus to the Bedford County Hospital.

ANOTHER score in favour of vaccination has been made in the Provinces of Bosnia and Herzegovina. Since 1880 vaccination has been systematically carried out with the result that Bosnia, formerly a hot-bed of small-pox, is now entirely free from it, and Herzegovina is practically free also.

The Dowager Empress of Russia has given about £11,000 to form a prize fund, the prizes to be awarded every fifth year, at the International Red Cross Conferences, to inventors of appliances and devices to ameliorate the condition of wounded and sick in times of war. The first awards—three in number—will be made in 1907. Information can be obtained from the British Red Cross Committee, 68 Victoria Street, S.W.

The Sanitary Congress in Paris is considering a project for the creation of an international sanitary bureau for the collection of information respecting infectious diseases, such as plague, cholera, and yellow fever, and also for the harmonious working of those sanitary regulations in the East which have so greatly contributed within the last five years to the preservation of public health as well as to the benefit of trade by the suppression of the old quarantine system. The international sanitary bureau would have its headquarters in Paris.

DR. SCHOFIELD calls attention in the *Times* to a serious error on the part of the South Western Railway, which it is to be hoped will meet with the earnest attention of the directors. It appears that the station clocks at Waterloo are kept in advance of Greenwich time, and trains very frequently depart before the advertised time. The complaint comes very appropriately from a member of the medical profession, for life or death may sometimes be decided by the possibility to secure prompt medical aid.

As matters now stand, it may be the fate of many medical men to strive their utmost to catch a train in response to an urgent telegram and to succeed in reaching the platform just before the advertised time of departure only to see the train disappearing in the distance. We endorse Dr. Schofield's complaint with no unfriendly feeling towards the South Western Railway, whose management of its main line traffic has our admiration. We recognise the efforts made to secure a punctual service for the arrival of trains, but if this is dependent on an unpunctual departure our admiration must be discounted.

